# D-Penicillamine Reveals the Amelioration of Seizure-Induced Neuronal Injury via Inhibiting Aqp11-Dependent Ferroptosis

**DOI:** 10.3390/antiox11081602

**Published:** 2022-08-19

**Authors:** Nan Yang, Kai Zhang, Qi-Wen Guan, Zhao-Jun Wang, Kang-Ni Chen, Xiao-Yuan Mao

**Affiliations:** 1 Department of Clinical Pharmacology, Xiangya Hospital, Central South University, 87 Xiangya Road, Changsha 410008, China; 2 Institute of Clinical Pharmacology, Hunan Key Laboratory of Pharmacogenetics, Central South University, 110 Xiangya Road, Changsha 410078, China; 3 Engineering Research Center of Applied Technology of Pharmacogenomics, Ministry of Education, 110 Xiangya Road, Changsha 410078, China; 4 National Clinical Research Center for Geriatric Disorders, Xiangya Hospital, Central South University, 87 Xiangya Road, Changsha 410008, China

**Keywords:** D-penicillamine, neuronal injury, ferroptosis, *Aqp11*, therapy

## Abstract

Repetitive seizures, a common phenomenon in diverse neurologic conditions such as epilepsy, can undoubtedly cause neuronal injury and our prior work reveals that ferroptosis is a contributing factor of neuronal damage post seizure. However, there is no drug available in clinical practice for ameliorating seizure-induced neuronal impairment via targeting ferroptosis. Our present work aimed to explore whether D-penicillamine (DPA), an originally approved drug for treating Wilson’s disease, inhibited neuronal ferroptosis and alleviated seizure-associated brain damage. Our findings revealed that DPA remarkably improved neuronal survival in kainic acid (KA)-treated mouse model. Furthermore, ferroptosis-associated indices including acyl-coA synthetase long chain family member 4 (ACSL4), prostaglandin-endoperoxide synthase 2 (*Ptgs2*) gene and lipid peroxide (LPO) level were significantly decreased in KA mouse model after DPA treatment. In a ferroptotic cell death model induced by glutamate or erastin, DPA was also validated to evidently suppress neuronal ferroptosis. The results from RNA-seq analysis indicated that *Aqp11*, a gene coding previously reported channel protein responsible for transporting water and small solutes, was identified as a molecular target by which DPA exerted anti-ferroptotic potential in neurons. The experimental results from in vivo *Aqp11* siRNA transfer into the brain also confirmed that knockdown of *Aqp11* abrogated the inhibitory effect of seizure-induced ferroptosis after DPA treatment, suggesting that the effects of DPA on ferroptosis process are dependent upon *Aqp11*. In conclusion, DPA can be repurposed to cure seizure disorders such as epilepsy.

## 1. Introduction

It is well established that seizure occurs in a variety of acute and chronic neurological disorders including neurodegenerative diseases, ischemic stroke, brain tumor and, especially, epilepsy characterized with abnormal and synchronous neuronal activity [1,2,3,4]. Nowadays, almost all the therapeutic interventions are applied for curing epileptic patients via controlling seizures [5]. Nevertheless, in epileptic conditions, there are still approximately 30% of patients who are not responsive to the current medications due to incomplete seizure control [6]. As such, it is of desperate need to explore novel therapeutic regime in order to completely cease seizure and finally improve the life quality. 

Repetitive seizures often activate a cell death-promoting mechanism and subsequently aggravate brain damage [7,8]. Ferroptosis is a novel type of regulatory cell death discovered in 2012 by the Stockwell laboratory [9]. Ferroptotic cells usually have the characteristics of iron-dependency and overproduction of lethal lipid peroxides. Our previous investigations delineate that ferroptosis are activated in multiple seizure rodent models induced by various chemical reagents such as kainic acid (KA), pentylenetetrazole, pilocarpine and ferric chloride [10,11,12,13]. Furthermore, the seizure activity and associated neuronal injury are remarkably prevented when treatment with ferroptosis inhibitors including ferrostatin-1 (Fer-1) or liproxstatin-1 (Lip-1), suggesting a promising approach in curing seizure disorders and protecting from brain injury via targeting ferroptosis. However, there is hitherto no ferroptosis-targeting drug available in clinical practice for curing seizure-associated neurological diseases, such as epilepsy.

D-penicillamine (DPA) is a prescription drug approved for treating patients with Wilson’s disease, primary biliary cirrhosis, rheumatoid arthritis or heavy metal poisoning in clinic as early as 1956 [14,15,16,17,18]. Previous investigations demonstrate that DPA has therapeutic potential possibly via copper chelation and inhibition of oxidative stress (OS) [19,20]. In addition, DPA has also been shown to abrogate the production of hydrogen peroxide and subsequent attenuate oxidative damage [21]. Since OS is always a contributing factor for triggering ferroptotsis via activation of lipid peroxidation [22], it is rational to speculate that DPA regulates ferroptosis and has a therapeutic potential against neuronal injury after seizure. Although previous investigations have revealed that the marked protection against pentylenetetrazole- or photic-induced seizures is found after treatment with DPA [23,24], whether the effects of DPA on seizure-induced brain injury and the potential mechanism are still not well characterized.

In the present work, we reported that DPA significantly ameliorates seizure-induced neuronal impairment in KA mouse model. Furthermore, ferroptosis is involved in the neuroprotection of DPA against KA-induced seizure in mice. In the ferroptosis death model induced by glutamate or erastin in HT22 neuronal cells, we also found that DPA dramatically abrogated ferroptosis-associated lipid peroxidation including decreased levels of lipid reactive oxygen species (ROS) and acyl-coA synthetase long chain family member 4 (ACSL4). Mechanistically, *Aqp11* was an identified molecular target underlying the inhibitory effect of DPA on neuronal ferroptosis process. In the vivo seizure mouse model by KA treatment, it was also confirmed that knock down of *Aqp11* by siRNA administration in mice brain significantly abrogated the inhibitory effect of seizure-induced ferroptosis after DPA treatment, suggesting that the inhibitory effect of DPA on ferroptosis process is dependent upon *Aqp11*. Altogether, our results show that *Aqp11*-dependent ferroptosis paradigm serves as a critical mechanism for the therapeutic potential of DPA against neuronal impairment post seizure. We also highlight that DPA can be repurposed for treating seizure disorders such as epilepsy. 

## 2. Materials and Methods

All the chemicals used in our present work were summarized in Table 1.

### 2.1. Preparation of KA-Induced Seizure Mouse Model and Drug Treatment

Male C57BL/6J mice (6–8 weeks of age, weighing 18–22 g) were provided by at the Centre for Animals of Central South University (Changsha, China). Prior to the experiment, mice were separately maintained in a constant environment with a 12 h light/12 h dark cycle, 22 ± 0.5 °C, 60 ± 10% humidity and other specific laboratory conditions. All animal experiments were conducted in strict accordance with principles presented in the National Institute of Health for the Care and Use of Laboratory Animals and approved by the Institutional Animal Ethics Committee of Central South University (approved number: 2020–0019). To prepare the seizure mouse model, the mice underwent the intrahippocampal injection of KA as described in our previous investigation [12,25]. For short, mice were anesthetized with sodium phenobarbital (50 mg/kg, i.p.) and carefully placed on a stereotaxic apparatus. Then, KA (1 μL, 250 ng/μL dissolved in saline) was stereotactically injected into the hippocampus according to the following coordinates: anteroposterior −2.0 mm; lateral −1.3 mm; dorsoventral −1.2 mm. After injection, the infusion needle was kept in place for 5–10 min to avoid liquid reflux. Mice in the control group underwent the same surgical procedure but received injection with an equal volume of phosphate buffered saline (PBS) instead of KA.

Experiment 1: To explore the optimal regime for the amelioration of DPA on seizure-induced neuronal injury, mice were randomly divided into six different groups as follows: (1) vehicle group (*n* = 6): stereotactical injection with 1 μL PBS after the intraperitoneal injection with physiological saline 2 h, and then injection with saline once a day for another three consecutive days; (2) KA group (*n* = 6): stereotactical injection with 1 μL KA (250 ng/µL) after the intraperitoneal injection with physiological saline 2 h, and then injection with saline once a day for another three consecutive days; (3) pre KA + DPA group (*n* = 6): stereotactical injection with 1 μL KA (250 ng/µL) after the intraperitoneal injection with 0.5 mg/kg DPA 2 h, then injection with saline once a day for another three consecutive days; (4) post KA + DPA group (*n* = 6): stereotactical injection with 1 μL KA (250 ng/µL) after the intraperitoneal injection with physiological saline 2 h, then injection with DPA once a day for another three consecutive days; (5) KA + DPA group (*n* = 6): stereotactical injection with 1 μL KA (250 ng/µL) after the intraperitoneal injection with 0.5 mg/kg DPA 2 h, then injection with DPA once a day for another three consecutive days; and (6) DPA group (*n* = 6): stereotactical injection with 1 μL PBS after the intraperitoneal injection with 0.5 mg/kg DPA 2 h, then injection with DPA once a day for another three consecutive days. The dose of DPA (0.5 mg/kg) was selected according to the prior report [24]. In addition, this dose is relevant to approximately 0.04 mg/kg in human, which is lower than the dose range of intake (8.3–25 mg/kg) according to the human effective dose formula as previously described [26]. It indicates that DPA at the dose of 0.5 mg/kg in mice is safe.

Experiment 2: To probe the inhibitory effect of DPA on the ferroptosis process following seizure-induced neuronal injury, the mice were randomly assigned into the four following groups: (1) vehicle group (*n* = 6): stereotactical injection with 1 μL PBS after the intraperitoneal injection with physiological saline 2 h, and then injection with saline once a day for another three consecutive days; (2) KA group (*n* = 6): stereotactical injection with 1 μL KA (250 ng/µL) after the intraperitoneal injection with physiological saline 2 h, and then injection with saline once a day for another three consecutive days; (3) pre KA + DPA group (*n* = 6): stereotactical injection with 1 μL KA (250 ng/µL) after the intraperitoneal injection with 0.5 mg/kg DPA 2 h, then injection with saline once a day for another three consecutive days; and (4) DPA group (*n* = 6): stereotactical injection with 1 μL PBS after the intraperitoneal injection with 0.5 mg/kg DPA 2 h, then injection with saline once a day for another three consecutive days.

### 2.2. Aqp11 siRNA Transfer in Mice Brain

*Aqp11* siRNA administration was carried out in the brain according to the previous investigations [27,28], in order to examine whether reduction of *Aqp11* may abrogate the inhibitory effect of DPA on neuronal ferroptosis post seizures. For short, mice were anesthetized with sodium phenobarbital (50 mg/kg, i.p.) and carefully placed on a stereotaxic apparatus. Then, 1 μL *Aqp11* siRNA or control siRNA (Ribobio, Guangzhou, China) was diluted with the same volume of in vivo transfection reagent (Entranster™-in vivo; Engreen, Beijing, China) and stereotactically injected into the hippocampus according to the following coordinates: anteroposterior −2.0 mm; lateral −1.3 mm; dorsoventral −1.2 mm. After injection for 48 h, the brains were rapidly isolated in an ice-cold saline. Hippocampus was carefully dissected for the subsequent detection.

### 2.3. Racine Score

Evaluation of seizure behavior was performed according to the Racine scale [29]. The standards of Racine stages were elaborated in the following description: stage 0, no response; stage 1, facial and whisker rhythmic twitching; stage 2, head bobbing and circling; stage 3, myoclonic and spasm in multiple limbs; stage 4, rearing and falling; stage 5, general tonic-clonic seizures with running and jumping; stage 6, death. Mice with the third or higher seizure stage were considered to be successfully kindled and enrolled in the subsequent experiment. 

### 2.4. Nissl Staining

Viable neurons were identified by detecting Nissl body with the Nissl stain solution. Briefly, brain tissue sections (10 μm) from each group were soaked in Nissl staining solution (C0117, Beyotime Biotechnology, Shanghai, China) for 10 min at room temperature. After dehydration, the brain slides were coverslipped with neutral balsam and imaged using an optical microscope (Leica, Wetzlar, Germany). Nissl bodies stained by Nissl solution from hippocampal CA1 and CA3 subregions were calculated by Image J software (Bethesda, MD, USA).

### 2.5. Fluoro-Jade B Assay

As previously described [25], degenerative neurons were recognized by Fluoro-Jade B (FJB) (AAT Bioquest, California, USA) staining. In brief, tissue slides (10 μm) were immersed 1% sodium hydroxide and 70% ethanol for 5 min and 2 min, respectively. Following three washes using distilled water, the slides were then incubated with 0.06% potassium permanganate for about 10 min. Following a 1–2 min of water rinse, tissue sections were then transferred for 20 min in a solution of FJB (0.0004%). Finally, slides were coverslipped with neutral balsam and viewed under a fluorescence microscope (Nikon, Tokyo, Japan). FJB-positive cells were calculated using Image J software (Bethesda, MD, USA).

### 2.6. Cell Culture

HT22 cell, an immortalized hippocampal cell line from mouse origin [30], which was provided by Shanghai Cell Bank of Chinese Academy of Sciences (Shanghai, China), was cultured in Dulbecco’s modified Eagle’s medium with high glucose supplemented with 10% fetal bovine serum (FBS) and 1% penicillin/streptomycin in an incubator with 5% CO_2_ atmosphere at 37 °C.

### 2.7. RNA-Seq Analysis

After drug treatments, RNA-seq analysis was carried out by OE Biotech Co., Ltd. (Shanghai, China). Purified RNA from three groups including control, glutamate-treated groups as well as combinational group (glutamate + DPA) was used for library construction with the Illumina TruSeq RNA Sample Prep Kit (FC-122–1001). The constructed library quality was checked using an Agilent 2100 Bioanalyzer (Agilent Technologies, Santa Clara, CA, USA). The read counts of each gene were subsequently acquired by htseq-count [31], and the fragments per kb per million reads (FPKM) value of each gene was calculated by Cufflinks [32]. Differentially expressed genes (DEGs) were obtained using the estimateSizeFactors and nbinomTest functions, as implemented in the DESeq (2012) R package [33]. The *p* value with less than 0.05 and fold-change more than 2 were set as the threshold to evaluate the significance of DEGs.

### 2.8. Real-Time Quantitative PCR

Total RNA from tissues or cells in each group was extracted using TRIzol reagent (Invitrogen). The quality of RNA was evaluated by the ratio of a 260/280 ratio in the range of 1.8–2.0 and a negative control was performed to confirm no DNA contamination in the RNA extraction. Total RNA (1 μg) was used for the synthesis of cDNA in a 20 μL reaction mixture using a PrimeScript™ RT reagent Kit (Takara). Then, 2 μL of cDNA was added in a 50 μL reaction mixture for quantitative PCR, which was carried out using a LC480 system (Roche) according to the following conditions: pre-denaturation at 95 °C for 30 s followed by 40 cycles of denaturation at 95 °C for 5 s, annealing at 60 °C for 30 s and elongation at 72 °C for 30 s. Six samples were run for each experimental group and each sample was detected in triplicate during the assay. The primer sequences were displayed in Table 2. Gene expressions were calculated using 2^−ΔΔCT^ method. 

### 2.9. Western Blot Assay

Total proteins were extracted from hippocampus tissues or cells using a cold lysis buffer (P0013, Beyotime Biotechnology Institute, Shanghai, China) with mixed protease and phosphatase inhibitors. A total of 20 μg proteins from different groups were separated by gel electrophoresis, shifted to polyvinylidene difluoride (PVDF) membranes (Millipore, Darmstadt, Germany) and blocked with 5% non-fat milk for 1 h at room temperature. The membranes were subsequently rinsed with primary antibodies including cleaved caspase-3, LC3II/I, ACSL4, cyclooxygenase-2 (COX-2), *Aqp11* while β-actin was used as control. Next day, the protein bands were probed with horseradish peroxidase (HRP) conjugated secondary anti-mouse or anti-rabbit antibodies for 1 h and then visualized using enhanced chemiluminescence kit. The imageJ software was utilized to analyze the significant difference in protein expression. Other details of antibodies were summarized in Table 3.

### 2.10. RNA Interference in HT22 Cell

Gene knockdown was assessed by RNA interference. SiRNA or matched control was supplied by RioBio Biotechnology (Guangzhou, China) and the target sequences were listed in Table 2. HT22 neuronal cells were transfected by adding mixtures including Lipofectamine™ RNAiMAX and siRNA. Following transfection for 48 h, cells were collected for Western blot analysis or cultivated in 24-well plates, which were treated with DPA and glutamate for the analysis of cell death.

### 2.11. Measurement of Lipid Peroxide (LPO) Levels

The LPO level of hippocampus tissues was determined by a Lipid Peroxidation kit (A106, Jiancheng Biotechnology, China). Cellular lipid ROS level was analyzed with a C11-BODIPY (581/591)-labelled method according to our previous investigation [12]. In brief, HT22 cells after drug treatments were trypsinized and incubated with C11-BODIPY (581/591) (2 μmol/L) for 15 min at 37 °C. Fluorescence signal was captured by a flow cytometer under 488 nm excitation wavelength. 

### 2.12. Immunofluorescence

Following transcardial perfusion with PBS, the whole brains from each group were carefully dissected. The brain tissue blocks were conducted cryo-sectioning at an average thickness of 8 µm using a cryostat (CM1900UV, Leica, Germany). The cryosections were then incubated with 0.2% Triton X-100 and 5% donkey serum in PBS. Afterward, sections were incubated with primary antibodies overnight. On the next day, the samples were washed and probed with the secondary antibody for 1 h at room temperature. The fluorescence microscope (Nikon, Tokyo, Japan) was employed to capture the fluorescence images. The detailed information of antibodies was indicated in Table 2.

### 2.13. Measurement of Copper Level in Mice Brain

Detection of copper level in various brain regions including hippocampus, frontal cortex and thalamus were carried out using a commercial Copper Colorimetric Assay Kit (E-BC-K300-M, Elabscience, Wuhan, China) as previously described [34]. Copper concentration was calculated by determination of optical density at the wavelength of 580 nm.

### 2.14. Statistical Analysis

All the data were presented as mean ± SEM. GraphPad 9.0 software (GraphPad, San Diego, CA, USA) was adopted for statistical analysis. The analysis of normal distribution was conducted using Shapiro–Wilk test. In terms of normally distributed data, statistical analysis methods include one-way ANOVA or repeated measure (RM)-two-way ANOVA with Tukey’s test and Sidak’s test. *p* value with less than 0.05 was considered statistically significant.

## 3. Results

### 3.1. DPA Ameliorates Seizure-Induced Neuronal Injury in KA-Treated Mouse Model

**Figure 1:**
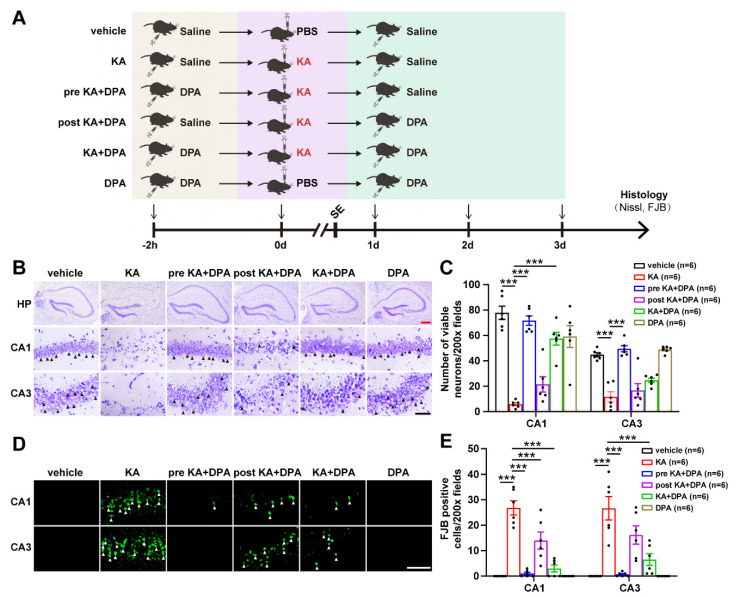
DPA ameliorates seizure-induced neuronal injury in KA-treated mouse model. (**A**) Experimental regimen for DPA (0.5 mg/kg) treatment. KA (250 ng/µL) was stereotactically injected to induce repetitive seizures (status epilepticus, marked with SE in this figure). Histological analysis including Nissl staining and FJB detection was carried out 3 d after KA or PBS injection. (**B**) Representative images by Nissl staining in the hippocampus showing the effects of DPA on viable neurons in KA-treated mice. Arrows indicate Nissl positive cells. Red scale bar indicates 200 μm; black scale bar indicates 50 μm. (**C**) Statistical analysis of Nissl staining results in hippocampal CA1 and CA3 subregions. (**D**) Representative images by FJB staining in the hippocampus showing the effects of DPA on the neurodegeneration in KA-treated mice. Arrows indicate FJB positive cells. Scale bar indicates 25 μm. (**E**) Statistical analysis of FJB staining results in hippocampal CA1 and CA3 subregions. All the data were expressed as mean ± SEM (*n* = 6). *** *p* < 0.001. Abbreviation: DPA, D-penicillamine; KA, kainic acid; SE, status epilepticus; PBS, phosphate buffered saline; FJB, Fluoro-Jade B; HP, hippocampus.

To explore whether neuronal injury post seizure was prevented after DPA treatment, seizure mouse model was prepared via intrahippocampal injection of KA and treated with DPA through three different intervention regimes as summarized in Figure 1A. In fact, prior to this work, we assessed the effects of DPA on seizures in mice. It was intriguing that treatment with DPA significantly alleviated the seizure behavior including generalized tonic-clonic seizures (Supplementary Movie). In addition, other indices for seizure evaluation, which include seizure score, number of seizures within 90 min and seizure duration [12], were also analyzed in our present work. It was found that DPA remarkably decreased the seizure score (Figure S1A), seizure frequency (number of seizures within 90 min in Figure S1B) and seizure duration (Figure S1C). Altogether, these data suggest the neuroprotection of DPA against KA-induced seizures in mice. We further explored the effects of DPA on seizure-induced neuronal injury via Nissl staining and FJB analysis. The results of Nissl staining indicated that intrahippocampal injection of KA significantly resulted in a decrease of viable neurons in hippocampal CA1 and CA3 subregions and treatment with DPA by three different intervention strategies all evidently protected neurons from damage with pre KA + DPA group (namely treatment with DPA 2 h prior to KA) showing the most pronounced protection (Figure 1C,D). Similarly, this group also had the optimal effect in preventing degenerated neurons in the CA1 and CA3 regions of hippocampus according to FJB staining results (Figure 1D,E). Taken together, these findings indicate that DPA exerts protection against seizure-induced neuronal injury.

### 3.2. Ferroptosis Is Involved in the Protection of DPA against Seizure-Related Neuronal Injury

**Figure 2:**
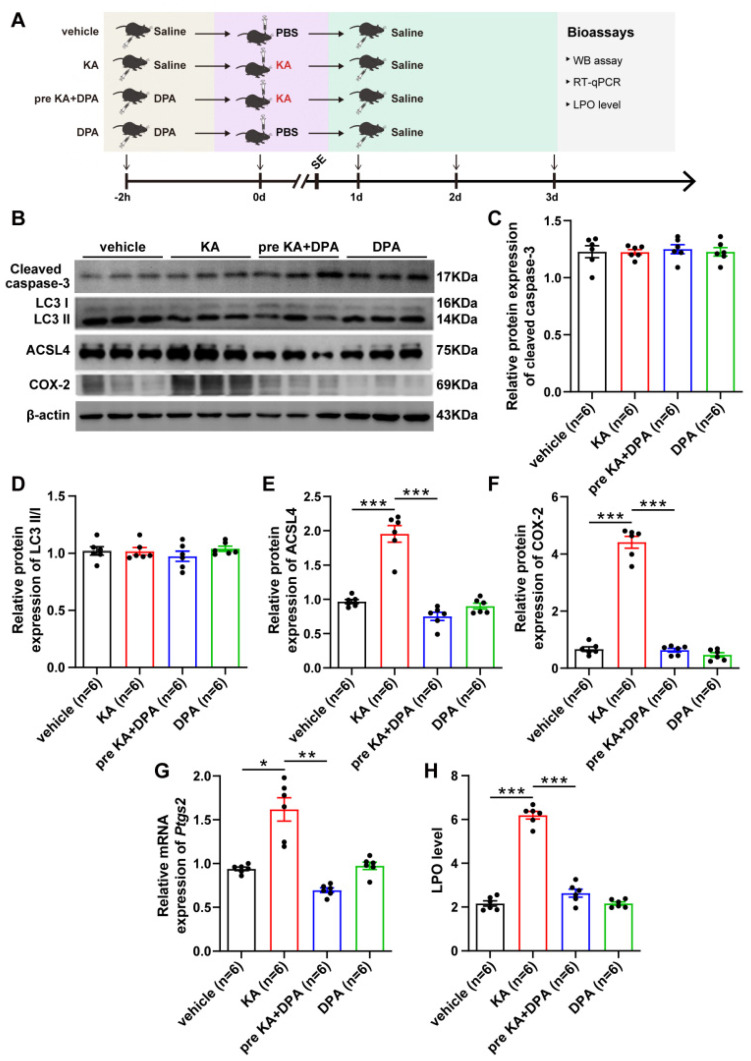
Ferroptosis is involved in the protection of DPA against KA-induced seizure mice. (**A**) Experimental scheme. Bioassays including WB, RT-qPCR and LPO detection were conducted 3 d after KA (250 ng/μL) or PBS injection. (**B**–**F**) Representative WB images and statistical analysis of protein expression in the hippocampus showing the effects of DPA on indices of apoptosis (cleaved caspase-3), autophagy (LC3II/I) and ferroptosis (ACSL4 and COX-2) in mice subjected to KA. (**G**,**H**) Effects of DPA on other ferroptosis indices including *Ptgs2* mRNA and LPO level in mice subjected to KA. All the data were expressed as mean ± SEM (*n* = 6). * *p* < 0.05, ** *p* < 0.01 and *** *p* < 0.001. Abbreviation: DPA, D-penicillamine; KA, kainic acid; SE, status epilepticus; PBS, phosphate buffered saline; LPO, lipid peroxide; RT-qPCR, real-time quantitative PCR; WB, Western blot; IF, immunofluorescence; ACSL4, acyl-coA synthetase long chain family member 4; COX-2, cyclooxygenase-2; *Ptgs2*, prostaglandin-endoperoxide synthase 2.

As DPA serves as a well-known copper-chelating agent [35,36], we firstly measured the copper level in KA-treated mice after DPA treatment. It was surprising that no significant difference was observed in hippocampus, frontal cortex and thalamus in mice subject to KA when treatment with DPA, compared with vehicle group (Figure S2), suggesting other molecular mechanism is involved in the protection of DPA against neuronal damage post seizure. Since multiple types of cell death modalities such as apoptosis, autophagy and ferroptosis appear in KA-induced seizure models [7], and these cell death modes are involved in seizure-induced neuronal injury. Therefore, we further probed whether the inhibitory effects of DPA on neuronal damage post seizures were associated with the regulation of cell death process. Western blot analysis was performed to evaluate the biomarkers of apoptosis (e.g., cleaved caspase-3), autophagy (e.g., LC3II/I) and ferroptosis (e.g., ACSL4 and COX-2) [25]. It was noted that DPA had an inhibitory effect on ferroptosis (shown by decreases of ACSL4 and COX-2 expression levels) while apoptotic cascade and autophagy were not evidently influenced in KA mouse model subjected to DPA treatment (Figure 2B–F). In addition, other ferroptotic indices including prostaglandin-endoperoxide synthase 2 (*Ptgs2*) mRNA and LPO level were also diminished after DPA treatment in comparison with vehicle-treated seizure group (Figure 2G,H). These data indicate that ferroptosis process is involved in the protection of DPA against seizure-induced neuronal impairment.

### 3.3. DPA Inhibits Glutamate-Induced Neuronal Ferroptosis In Vitro

**Figure 3:**
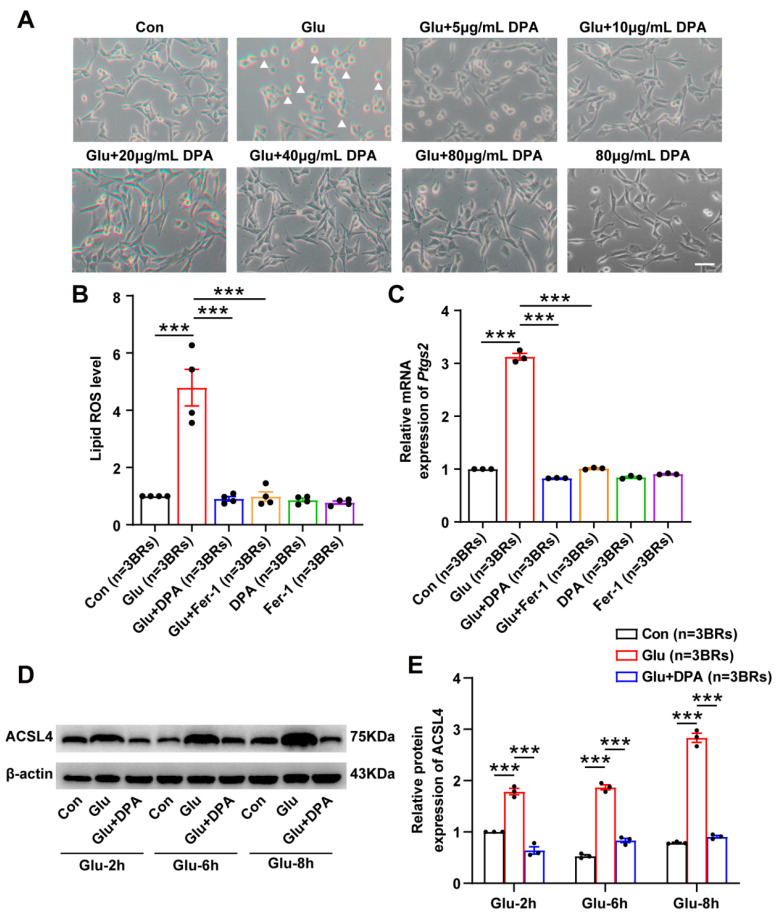
DPA inhibits glutamate-induced neuronal ferroptosis in vitro. (**A**) Representative phase contrast images showing the effects of DPA by different concentrations (5, 10, 20, 40 and 80 μg/mL) on neuronal ferroptosis induced by 5 mmol/L of glutamate for 8 h in HT22 cells. DPA was treated 2 h prior to glutamate treatment for 8 h in HT22 cells. The optimal concentration of DPA for the inhibition of neuronal ferroptosis in HT22 cells after glutamate exposure was 10 μg/mL, which was employed for the subsequent experiments. Arrows indicate the dead neurons. Scale bar indicates 50 μm. (**B**–**E**) The effects of DPA on ferroptosis-associated indices including lipid ROS, *Ptgs2* mRNA and protein expression levels of ACSL4 in glutamate-induced ferroptosis in HT22 neuronal cells. The concentrations of DPA and Fer-1 (a specific ferroptosis inhibitor) were 10 μg/mL and 12.5 μmol/L, respectively. Regarding the detections of lipid ROS and *Ptgs2* mRNA, HT22 cells were treated with DPA or Fer-1 2 h prior to glutamate exposure for 8 h while the cell samples were collected for determination of ACSL4 protein expression following pretreatment with DPA for 2 h and glutamate challenge at different time points (2 h, 6 h and 8 h). All the data were expressed as mean ± SEM (*n* = 3 independent biological replicates). *** *p* < 0.001. Abbreviation: Glu, glutamate; DPA, D-penicillamine; Fer-1, ferrostatin-1; *Ptgs2*, prostaglandin-endoperoxide synthase 2; lipid ROS, lipid reactive oxygen species; ACSL4, acyl-coA synthetase long chain family member 4; BRs, biological replicates.

Glutamate is a well-known excitatory neurotransmitter and its accumulation in the brain is a contributing factor for seizure generation [37]. Moreover, our prior work confirmed that glutamate by the concentration of 5 mM for 8 h was sufficient for triggering ferroptosis in HT22 cells [12,25]. Thus, glutamate-induced ferroptotic cell death model in HT22 neuronal cell line was selected in our present work to further clarify the molecular mechanism underlying the inhibition of DPA on neuronal ferroptosis. As shown in Figure 3A, HT22 cells challenged with 5 mM glutamate for 8 h exhibited massive cell death. However, treatment with different concentrations of DPA (5, 10, 20, 40 and 80 μg/mL) significantly increased viable neuronal cells notable 10 μg/mL. The inhibition of DPA on neuronal ferroptosis in vitro were also observed since the indices of lipid peroxidation including lipid ROS level (Figure 3B) and the protein expression of ACSL4 (Figure 3D) and other molecular marker such as *Ptgs2* mRNA (Figure 3C) were remarkably suppressed in glutamate-induced ferroptosis model after DPA treatment. Taken together, these data suggest that DPA is able to inhibit glutamate-induced neuronal ferroptosis. 

### 3.4. DPA Inhibits Erastin-Induced Neuronal Ferroptosis In Vitro

**Figure 4:**
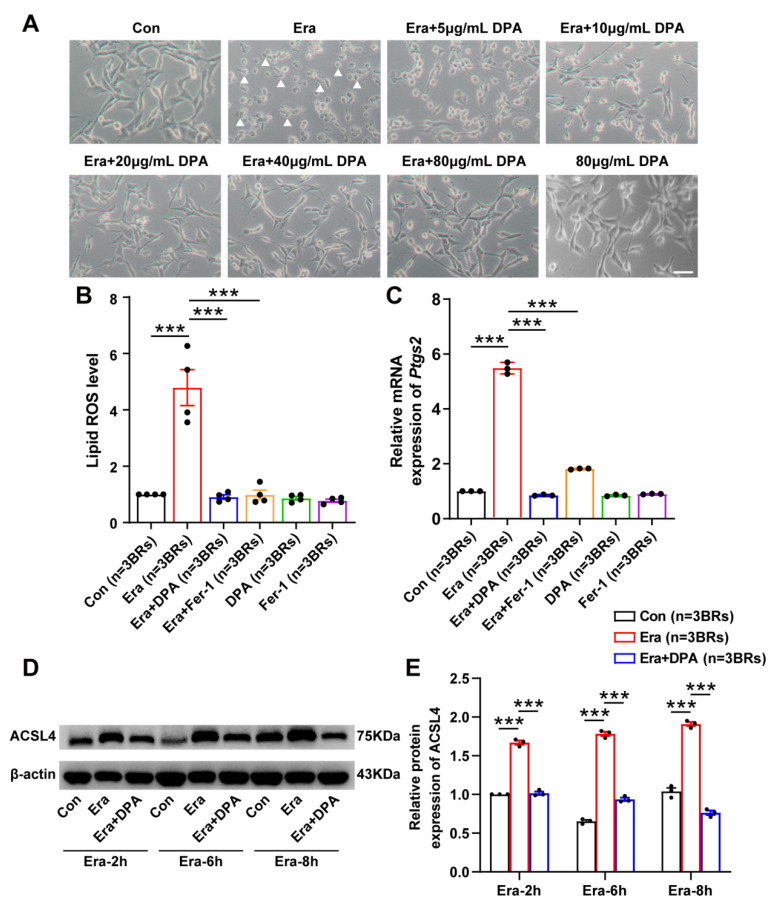
The anti-ferroptosis effect of DPA is validated in erastin-induced neuronal ferroptosis in vitro. (**A**) Representative phase contrast images indicating the effects of DPA by different concentrations (5, 10, 20, 40 and 80 μg/mL) on neuronal ferroptosis induced by erastin (0.5 μmol/L) for 8 h in HT22 cells. DPA was incubated 2 h prior to erastin challenge for 8 h in HT22 cells. DPA at the concentration of 20 μg/mL exerted the most obvious protection against ferroptotic cell death in HT22 following erastin exposure. Thus, this concentration was utilized for the subsequent experiments. Arrows indicate the dead neurons. Scale bar indicates 50 μm. (**B**–**E**) The effects of DPA on ferroptosis-associated indices including lipid ROS, *Ptgs2* mRNA and protein expression levels of ACSL4 in erastin-induced ferroptosis in HT22 neuronal cells. The concentrations of DPA and Fer-1 (a specific ferroptosis inhibitor) in HT22 cells were 20 μg/mL and 12.5 μmol/L, respectively. In terms of the detections of lipid ROS and *Ptgs2* mRNA, HT22 cells were treated with DPA or Fer-1 2 h prior to erastin treatment for 8 h while the cell samples used for detecting ACSL4 protein expression were collected following pretreatment with DPA for 2 h and erastin exposure at different time points (2 h, 6 h and 8 h). All the data were expressed as mean ± SEM (*n* = 3 independent biological replicates). *** *p* < 0.001. Abbreviation: Era, erastin; DPA, D-penicillamine; Fer-1, ferrostatin-1; *Ptgs2*, prostaglandin-endoperoxide synthase 2; lipid ROS, lipid reactive oxygen species; ACSL4, acyl-coA synthetase long chain family member 4; BRs, biological replicates.

Next, we further validated the anti-ferroptosis potential of DPA in ferroptotic cell death model triggered by the specific ferroptosis-inducing reagent erastin. In this model, DPA was found to significantly diminish the proportion of cell death by different concentrations of DPA (5, 10, 20, 40 and 80 μg/mL) notable 20 μg/mL (Figure 4A). Other indices for assessing ferroptosis process including lipid ROS, the protein expression level of ACSL4 [38,39] and the mRNA expression of *Ptgs2* [40] were also detected to explore the ferroptosis-inhibiting effect of DPA in neurons. It was found that DPA remarkably caused the downregulation of lipid ROS (Figure 4B), and decreased the protein expression of ACSL4 in erastin-induced ferroptosis in HT22 cells (Figure 4D). The results by real-time quantitative PCR analysis also revealed the decrease of *Ptgs2* mRNA (Figure 4C) in this ferroptotic cell death model after DPA treatment. Collectively, these results together with those in glutamate-induced cell death model indicate that DPA indeed has an inhibitory effect on neuronal ferroptosis.

### 3.5. Aqp11 Is a Key Target Involved in the Protection of DPA against Ferroptosis In Vitro

**Figure 5:**
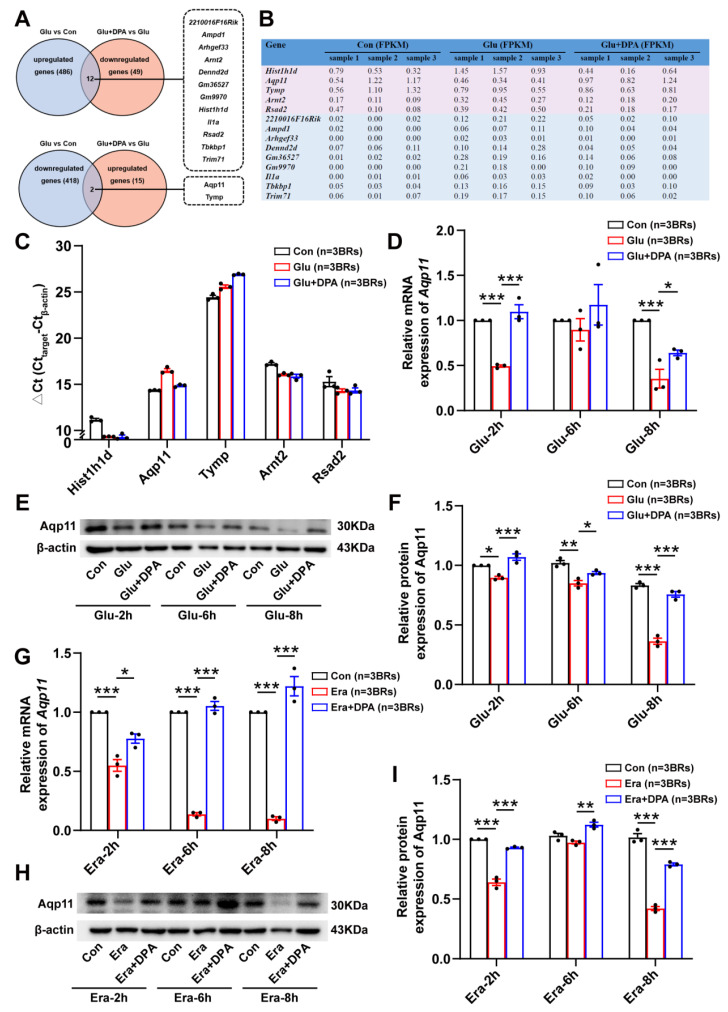
*Aqp11* is a key target involved in the protection of DPA against ferroptosis in vitro. (**A**) Summary of differentially expressed genes in glutamate-induced neuronal ferroptosis in HT22 cells following DPA treatment. Cell samples were collected for RNA-seq analysis after DPA pretreatment with 10 μg/mL for 2 h and glutamate challenge (5 mmol/L, 8 h). Totally, 14 differentially expressed genes or pseudogenes were screened, in which 12 genes or pseudogenes were up-regulated in glutamate group and were down-regulated in the combinational treatment with glutamate and DPA, while other 2 genes were down-regulated in glutamate group and were up-regulated in the combinational treatment with glutamate and DPA. (**B**) Detailed information of fragments per kilobase per million mapped reads (FPKM) obtained from RNA sequencing of 14 genes. (**C**) Verification of five genes with high FPKM values including *Hist1h1d*, *Aqp11*, *Tymp*, *Arnt2* and *Rsad2* by RT-qPCR. Cell samples were collected after DPA pretreatment with 10 μg/mL for 2 h and glutamate challenge (5 mmol/L, 8 h). (**D**–**F**) Effects of DPA pretreatment with 10 μg/mL for 2 h on *Aqp11* mRNA and protein expressions in HT22 neuronal cells following glutamate challenge (5 mmol/L) at different time points (2 h, 6 h and 8 h). (**G**–**I**) Effects of DPA pretreatment with 20 μg/mL for 2 h on *Aqp11* mRNA and protein expressions in HT22 neuronal cells following erastin challenge (0.5 μmol/L) at different time points (2 h, 6 h and 8 h). All the data were expressed as mean ± SEM (*n* = 3 independent biological replicates). * *p* < 0.05, ** *p* < 0.01 and *** *p* < 0.001. Abbreviation: DPA, D-penicillamine; Glu, glutamate; Era, erastin; FPKM, fragments per kb per million reads; BRs, biological replicates.

To further probe the molecular target by which DPA abrogated neuronal ferroptosis, RNA-seq analysis was employed in our present study. It was found that there are 14 differentially expressed genes in total, among which 12 genes were up-regulated in glutamate-treated group and down-regulated in glutamate-induced ferroptotic cell death group treated with DPA, while 2 genes were down-regulated in glutamate group and up-regulated in HT22 cells treated with glutamate and DPA (Figure 5A). The FPKM values of these genes were also summarized in Figure 5B. Some genes including *2210016F16Rik*, *Ampd1*, *Arhgef33*, *Denn2d*, *Gm36527*, *Gm9970*, *Il1α*, *Tbkbp1* and *Trim71* were excluded for further validation due to the low FPKM values in three independent replicates. After validation of differentially expressed genes including *Hist1h1d*, *Aqp11*, *Tymp*, *Arnt2* and *Rsad2*, it was interesting that only the Ct value of *Aqp11* was confirmed to increase in glutamate-treated group and decrease in the group treated with glutamate and DPA, which corresponded the decrease of gene expression in HT22 cells following glutamate stimulation and elevation of gene expression after treatment with glutamate and DPA, respectively (Figure 5C). Following glutamate treatment at different time points (2 h, 6 h and 8 h), DPA was also found to increase expressions of *Aqp11* at mRNA and protein levels (Figure 5D–F). Consistently, in HT22 cells treated with the specific neuronal ferroptosis-inducer erastin at different time points (2 h, 6 h and 8 h), it was also found that DPA increased the mRNA and protein expressions of *Aqp11* (Figure 5G–I). Collectively, these results suggest that *Aqp11* is identified to be involved in the ferroptosis-inhibition effect of DPA on neurons. 

### 3.6. DPA Increases the Expression and Distribution of Aqp11 in KA-Induced Seizures in Mice

**Figure 6:**
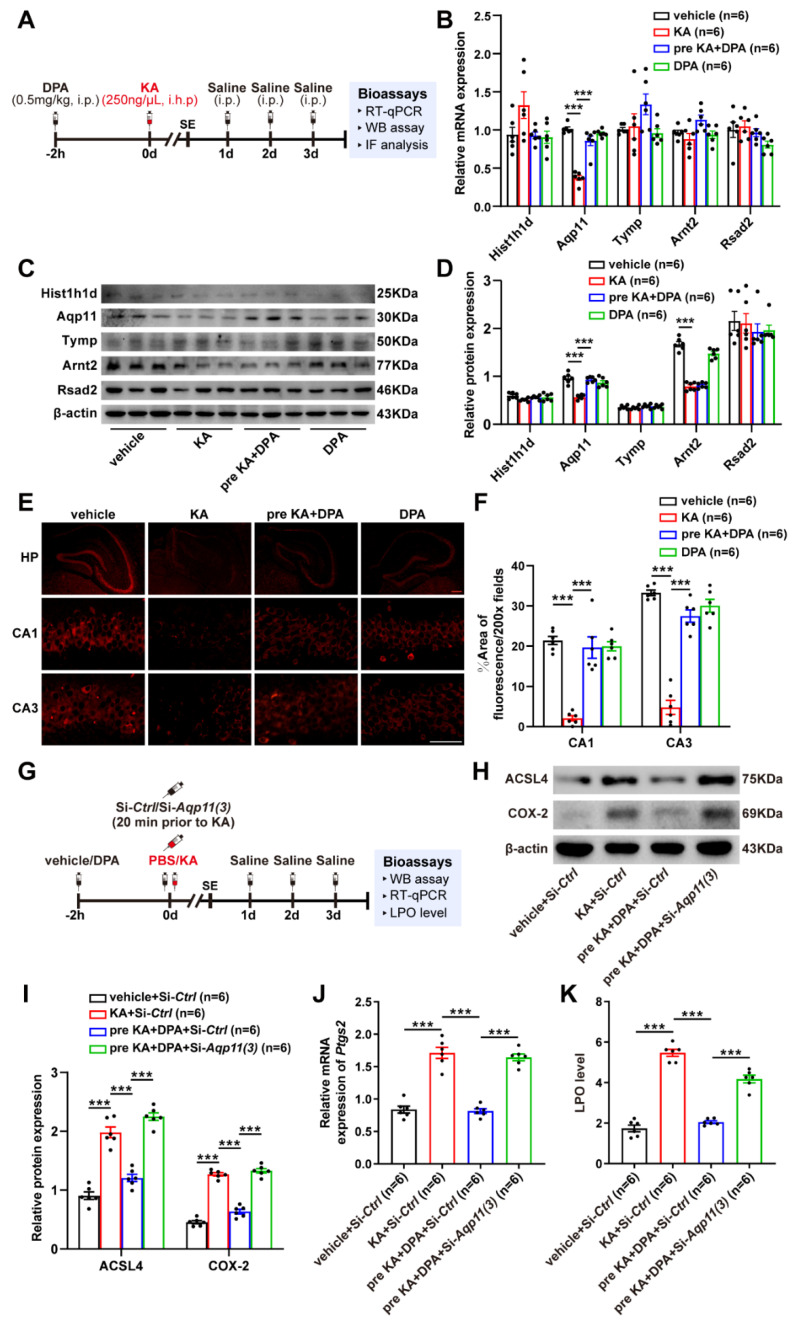
DPA attenuates KA-induced seizures via inhibiting *Aqp11*-dependent ferroptosis. (**A**) Experimental timeline. Bioassays including RT-qPCR, WB, IF and detection of LPO level were carried out 3 d after KA (250 ng/μL) or PBS injection. (**B**,**C**) Effects of DPA (0.5 mg/kg) on the mRNA and protein expressions of Hist1h1d, *Aqp11*, Tymp, Arnt2 and Rsad2 in KA-treated mice by RT-qPCR and WB, respectively. (**D**,**E**) Effects of DPA (0.5 mg/kg) on the protein distribution of *Aqp11* in KA-treated mice. Red scale bar indicated 200 μm; white scale bar indicated 50 μm. (**F**) Schematic description of si-*Aqp11* transfer in mice brain following KA-induced seizure and DPA treatment. Hippocampal tissue samples were collected for detection of ferroptosis-associated indices (ACSL4 and COX-2) 3 d after KA (250 ng/μL) or PBS injection. (**G**–**K**) Role of knock down of *Aqp11* by siRNA administration in vivo on DPA’s effects on the protein expressions of ACSL4 and COX-2 as well as mRNA expression of *Ptgs2* together with LPO level following KA-induced seizure in mice. All the data were expressed as mean ± SEM (*n* = 6). *** *p* < 0.001. Abbreviation: DPA, D-penicillamine; KA, kainic acid; SE, status epilepticus; RT-qPCR, real-time quantitative PCR; WB, Western blot; IF, immunofluorescence; LPO, lipid peroxide; HP, hippocampus; PBS, phosphate buffered saline; ACSL4, acyl-coA synthetase long chain family member 4; COX-2, cyclooxygenase-2; *Ptgs2*, prostaglandin-endoperoxide synthase 2.

Next, the screened targets in vitro (Hist1h1d, *Aqp11*, Tymp, Arnt2 and Rsad2) with high FPKM value were further validated in KA-induced mouse seizures at mRNA and protein levels. The experimental scheme was summarized as shown in Figure 6A. It was noted that only *Aqp11* was found to decrease in seizure mouse model and treatment with DPA significantly prevented this phenomenon (Figure 6B–D), which was consistent with the results shown in neuronal ferroptosis model induced by glutamate or erastin. Anyways, the results of immunofluorescence also revealed that there are evident decreases of *Aqp11* protein distributions in hippocampus especially CA1 and CA3 subregions in seizure mouse model (Figure 6E,F). Nevertheless, treatment with DPA in seizure mouse efficiently increased the protein distribution of *Aqp11*. In order to further explore whether the inhibitory effects of DPA on ferroptosis was dependent upon *Aqp11*, since there is no specific *Aqp11* inhibitor available, in vivo siRNA administration was conducted to knockdown *Aqp11* as summarized in Figure 6G. Prior to conduction of this experiment, three pieces of siRNA sequences for *Aqp11* were designed and the knockdown efficiency was validated via detecting the protein expression of *Aqp11*. It was obvious that only si-*Aqp11* (3) transfection in HT22 cell for 48 h exhibited nearly 70% reduction of *Aqp11* protein level (Figure S3). Knockdown of *Aqp11* by siRNA (3) significantly prevented the protection of DPA against glutamate-induced cell death (Figure S4). Therefore, si-*Aqp11* (3) was selected for the subsequent in vivo transfection. Indeed, in vivo transfection of si-*Aqp11* also resulted in the remarkable decrease of *Aqp11* protein level (Figure S5). It was intriguing that knockdown of *Aqp11* abrogated the reductions of ferroptosis-associated indices (the protein expressions of ACSL4 and COX-2 as well as mRNA expression of *Ptgs2* together with LPO level) in KA-treated mice after DPA treatment (Figure 6H–K). Altogether, these results indicate that DPA suppresses the ferroptosis process via activation of *Aqp11* in KA-induced seizure model.

## 4. Discussion

**Figure 7:**
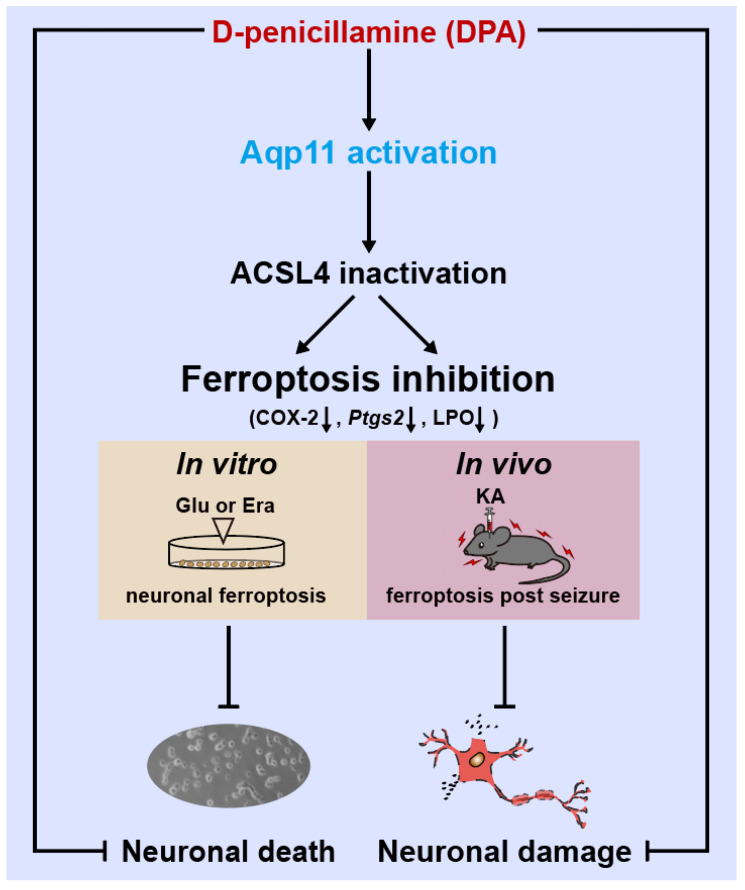
Schematic diagram illustrating DPA alleviates ferroptosis process and abrogates seizure-induced neuronal damage via activation of *Aqp11*. Abbreviation: ACSL4, acyl-coA synthetase long chain family member 4; COX-2, cyclooxygenase-2; *Ptgs2*, prostaglandin-endoperoxide synthase 2; LPO, lipid peroxide; Glu, glutamate; Era, erastin; KA, kainic acid.

The present work focused on the effects of DPA on seizure-induced neuronal injury and its potential molecular mechanism. The major findings of the study are shown as follows: (1) DPA exerts the pronounced protection against seizure-induced neuronal impairment; (2) ferroptosis is involved in the amelioration of DPA on the injured neurons post seizure; (3) *Aqp11* is identified to be a key molecular target for the inhibitory effect of DPA on neuronal ferroptosis in vitro and on seizure-induced neuronal damage in vivo (Figure 7).

DPA is a first-line drug for treating Wilson’s disease in clinical practice, and, also, has wide therapeutic applications in curing chronic active hepatitis, rheumatoid arthritis, systemic sclerosis, primary biliary cirrhosis and so on [41,42,43,44,45]. Previously, DPA has been demonstrated to mitigate the neurotoxicity caused by heavy metal thallium [46]. It has been reported that thallium poisoning can lead to neurological abnormalities that affect lower limb motor function [46,47]. Combinational treatment with DPA and Prussian blue significantly prevents the dysfunction of purkinje cells in the brain of thallium poisoned rats. These data indicate the alleviation of neurotoxicity after DPA treatment. In addition, DPA is also found to decrease the serum levels of copper and OS although results are still inconclusive regarding the efficacy of DPA on the rate of cognitive decline in Alzheimer’s disease (AD) patients [48]. Delivery of DPA into the brain via nanoparticle-based technique has also shown to evidently prevent Aβ accumulation in AD and reduce metal ion accumulation in other central nervous system (CNS) diseases such as Parkinson’s disease (PD) [49]. These findings support the notion that DPA has neuroprotective potential. The results from our present work reveal for the first time that DPA has remarkable neuroprotection against seizure-induced neuronal impairment, which suggest that DPA can be repurposed to cure seizure disorders such as epilepsy. Rahimi found that DPA at the dose of 0.5 mg/kg had anticonvulsant effects in pentylenetetrazole-induced seizures in mice [24], which was partly consistent with our present study.

Our findings also provide the evidence supporting that ferroptosis is involved in the amelioration of DPA on seizure-induced neuronal injury. Brain damage has been extensively reported in the seizure generation. In the early work demonstrated by Olney [50], systemic administration or intra-amygdaloid injection of KA in rats induces limbic seizures and subsequently develop acute brain damage in limbic region. The consistent findings are also found in Ben-Ari’s study [51]. It is firmly established that prolonged seizures (e.g., status epilepticus) can cause injury to the brain [52]. The hippocampal damage post seizure has also been demonstrated to increase the occurrence of recurrent seizures [8]. Our previous investigation showing ferroptosis is involved in seizure-induced neuronal injury [25] indicates that targeting ferroptosis plays a critical role for attenuating seizure-associated brain damage and seizure generation. In the present study, we demonstrate that DPA alleviates neuronal impairment post KA-induced seizure and suppresses ferroptosis-related lipid peroxidation. A previous investigation deciphered that treatment with DPA had a significant inhibition of the lipid peroxidative process in the iron-injected rat brain cortex [53], which is in line with our findings. 

We further identify *Aqp11* is a molecular target underlying the inhibitory effects of DPA on neuronal ferroptosis via RNA-seq analysis. *Aqp11* is an intracellular aquaporin whose original biological function is transporting water and small solutes [54]. It has wide distribution in a variety of organs including testis, thymus, kidney, liver, intestine, heart and brain [55,56,57]. Regarding the distribution of *Aqp11* in the brain, previous investigations reveal that *Aqp11* mainly localizes in the dendrites of Purkinje cells, hippocampal neurons, cerebral cortical neurons, the epithelium of the choroid plexus and the endothelium of brain capillaries [55,58]. Apart from serving as a kind of water channel protein, *Aqp11* has also been demonstrated to possess other biological functions. For instance, the localization of *Aqp11* in the epithelium supports that it is involved in the permeability of the blood-brain-barrier (BBB) and the pathophysiology of brain edema. Indeed, Xi demonstrated that *Aqp11* was up-regulated following intracerebral hemorrhage (ICH), which was accompanied with increased BBB permeability and development of brain edema [59]. Our present work revealed that *Aqp11* was down-regulated in KA-induced seizure model and ferroptotic neuronal death model. Furthermore, the up-regulation of *Aqp11* in our current work was involved in the inhibitory effects of DPA on seizure-induced neuronal injury and ferroptosis process. Knock down of *Aqp11* by in vivo siRNA transfer also remarkably abrogated the anti-ferroptosis effect of DPA on KA-induced seizure in mice, suggesting that the effects of DPA on ferroptosis process are dependent upon *Aqp11*. In fact, the counteracting effect of *Aqp11* on OS (a critical factor contributing to ferroptosis) has also been reported by Hoshino’s work showing that mice deficiency of *Aqp11* a dramatic increase of OS in the kidney [60], which is in part consistent with our results. It is rational to speculate that the regulatory role of *Aqp11* on the neuronal ferroptosis found in our present work may be associated with oxygen permeability, finally leading to oxidative stress. However, further investigation is indispensable to clarify this issue. In addition, it is also noted that the biological function of *Aqp11* in seizure model in our present work is completely distinct from the results reported in the ICH as mentioned above. That is to say, the inhibitory role of Aqp11 in seizure-induced neuronal injury and ferroptosis in our present work while the detrimental effect of *Aqp11* in ICH. The discrepancy may be attributable to the different disease contexts. It also suggests that *Aqp11* has distinct functions depending on specific disease models. Taken together, our present work discloses novel biological functions, namely, protection of neuronal impairment post seizure and anti-ferroptosis potential. 

There are also some items required to be clarified in the future. First, the detection of DPA level in the brain tissue is essential in order to ascertain whether it is able to cross the blood-brain-barrier. Second, from a translational perspective, the safety of DPA under seizure conditions is essential to be assessed as previous investigations have revealed the hepatic toxicity [61], decreased serum alanine aminotransferase activity [62] and bronchiolitis obliterans [63] following treatment with DPA in patients. Third, the molecular event by which *Aqp11* regulates neuronal ferroptosis process is still not completely concrete. Forth, whether a direct interaction of *Aqp11* and DPA exist is also essential to be clarified in the future investigation. Anyway, our findings showing the evidently inhibitory effect of DPA on neuronal impairment post seizure undoubtedly hold promise for providing the potential therapeutic evidence for patients with seizures.

## 5. Conclusions

In summary, our findings reveal that DPA efficiently alleviates seizure-induced neuronal injury and furthermore *Aqp11*-dependent ferroptosis inhibition is involved in DPA’s neuroprotection. We believe that D-penicillamine is a promising agent for the treatment of seizure disorders such as epilepsy.

## Figures and Tables

**Table 1:** Chemicals used in our present study.

Chemical	Source	Identifier
Kainic acid	Sigma	K0250, more than 99% purity
D-penicillamine	Sigma	P4875, 98–101% purity
Glutamate	Sigma	G8415, 98.5–100.5% purity
Erastin	Selleck	S7242
Ferrostatin-1	Selleck	S7243
Dimethyl sulfoxide	Sigma	D2650, more than 99.7% purity
Dulbecco’s modified Eagle’s medium	Gibco	C11995500BT
Hank’s Balanced Salt Solution	Gibco	14065056BT
Fetal bovine serum	Gibco	10099–141
Penicillin-Streptomycin	Gibco	15140122
Lipid peroxidase kit	Jiancheng Biotechnology	A106
Copper Colorimetric Assay Kit	Elabscience	E-BC-K300-M
BODIPY^TM^ 581/591 C11	Invitrogen	D3861
Illumina TruSeq RNA Sample Prep Kit	Illumina	FC-122-1001
TRIzol	Invitrogen	15596026
PrimeScript^TM^ RT reagent Kit with gDNA Eraser	TAKARA	RR047A
cDNA synthesis kit	TAKARA	RR820A
Diethypyrocarbonate-treated Water	Beyotime Biotechnology	R0021
Lysis buffer	Beyotime Biotechnology	P0013
ECL prime Western blotting det	GE	RPN2232
Triton X-100 solution	Beyotime Biotechnology	ST797
Phosphate-buffered saline	Solarbio	P1010
Normal donkey serum	Solarbio	SL050
Fluoro-Jade B	AAT Bioquest	23061
Nissl staining solution	Beyotime Biotechnology	C0117
Lipofectamine^TM^ RNAiMAX	Thermo	13778075
Entranster^TM^-in vivo	Engreen Biosystem	18668-11-1

**Table 2:** The primer sequences used in the present study.

**siRNA Sequences**			
**Name/Gene ID**	**Nomenclature in Our Paper**	**Target Sequences (5′-3′)**	
*Aqp11* (66333)	*Si-Aqp11 (1)*	CTCTGACACTGATCTACTT	
	*Si-Aqp11 (2)*	CAAGTACCATTACGACGAA	
	*Si-Aqp11 (3)*	CTTCCATGGCTGCATAACA	
**RT-qPCR primers**			
**Name/Gene ID**	**Oligonucleotide**	**Primer sequences (5′-3′)**	**Product size (bp)**
*Ptgs2* (19225)	Upper primer	GGGAGTCTGGAACATTGTGAA	112
	Lower primer	GTGCACATTGTAAGTAGGTGGACT	
*Hist1h1d* (14957)	Upper primer	GTGGAGAAGACACCTGTGAAG	535
	Lower primer	CCTTGGCTGGACTCTTTGCT	
*Aqp11* (66333)	Upper primer	TGGGGCTAATGCTGCTGTTC	300
	Lower primer	CACCCATTTCGGGGGACATA	
*Tymp* (72962)	Upper primer	CGCGGTGATAGATGGAAGAGC	187
	Lower primer	CACACCTCCTGTGGAGTGTT	
*Arnt2* (11864)	Upper primer	TTATCACGTTTGTGGACCCCA	269
	Lower primer	GTTGGTGCAGGTGACGTACT	
*Rsad2* (58185)	Upper primer	TGCTGGCTGAGAATAGCATTAGG	112
	Lower primer	GCTGAGTGCTGTTCCCATCT	
*β-actin* (11461)	Upper primer	GTGACGTTGACATCCGTAAAGA	245
	Lower primer	GCCGGACTCATCGTACTCC	

**Table 3:** Antibodies used in the present study.

Application	Antibody	Species	Dilution	Cat. No.	Source
Western blot (Primary antibodies)	Cleaved caspase-3 LC3II/I	mouse rabbit	1:500 1:1000	sc-373730 4108S	SantaCruz Cell Signaling Technology
	ACSL4	mouse	1:500	sc-365230	SantaCruz
	COX-2	rabbit	1:3000	A5787	Abclonal
	Aqp11	rabbit	1:2000	AP5805b	Abgent
	Hist1h1d	rabbit	1:1000	12177-1	Absci
	Tymp	rabbit	1:1000	12383-1-AP	Ptglab
	Arnt2	rabbit	1:1000	A8060	Abclonal
	Rsad2	rabbit	1:1000	A8271	Abclonal
	β-actin	rabbit	1:10,000	AP0060	Bioworld
Immunofluorescence (Primary antibodies)	Aqp11	rabbit	1:500	AP5805b	Abgent
Western blot (Secondary antibodies)	Goat anti-mouse IgG HRP Goat anti-rabbit IgG HRP	goat goat	1:10,000 1:10,000	ab97023 A9169	Abcam Sigma
Immunofluorescence (Secondary antibodies)	Alexa Fluor^®^488 Donkey anti-Rabbit IgG (H + L)	donkey	1:250	A-21206	Thermo

## Data Availability

All of the data contained within the article and Supplementary Materials.

## Supplementary Materials

The following supporting information can be downloaded at: https://www.mdpi.com/article/10.3390/antiox11081602/s1, Supplementary Movie. Figure S1: DPA protects against KA-induced seizures in mice. Figure S2: DPA does not alter the copper level in the brain tissues of KA-treated mice. Figure S3: Si-*Aqp11 (3)* evidently decreases the protein expression of *Aqp11* in HT22 cell. Figure S4: Knockdown of *Aqp11* by siRNA transfection significantly blocks the protection of DPA against glutamate-induced cell death in HT22 cells. Figure S5: Si-*Aqp11 (3)* administration in vivo significantly results in the reduction of Aqp11 protein expression in mice hippocampus.

## Abbreviations

ACSL4, acyl-coA synthetase long chain family member 4; AD, Alzheimer’s disease; BRs, biological replicates; BBB, blood-brain-barrier; COX-2, cyclooxygenase-2; CNS, central nervous system; DPA, D-penicillamine; DEGs, differentially expressed genes; Era, erastin; Fer-1, ferrostatin-1; FJB, Fluoro-Jade B; FBS, fetal bovine serum; FPKM, fragments per kb per million reads; Glu, glutamate; HRP, horseradish peroxidase; HP, hippocampus; ICH, intracerebral hemorrhage; IF, immunofluorescence; KA, kainic acid; Lip-1, liproxstatin-1; LPO, lipid peroxide; OS, oxidative stress; PBS, phosphate buffered saline; PVDF, polyvinylidene difluoride; Ptgs2, prostaglandin-endoperoxide synthase 2; PD, Parkinson’s disease; ROS, reactive oxygen species; RM, repeated measure; RT-qPCR, real-time quantitative PCR; SE, status epilepticus; WB, Western blot.
