# Supplementary material for: D-Penicillamine Reveals the Amelioration of Seizure-Induced Neuronal Injury via Inhibiting Aqp11-Dependent Ferroptosis
Source: Antioxidants (Basel). 2022 Aug 19;11(8):1602. doi: 10.3390/antiox11081602 (PMC9405105; doi:10.3390/antiox11081602)
Supplement: Supplementary file 1 [file antioxidants-11-01602-s001.zip › antioxidants-1877441--supplementary/antioxidants-1877441--supplementary.pdf]

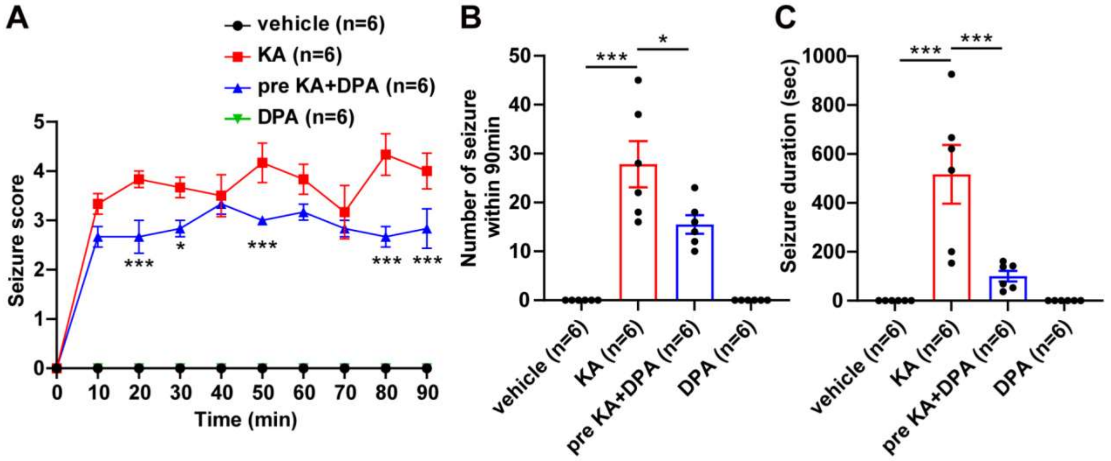

Figure S1. DPA protects against KA-induced seizures in mice. Seizure evaluation was conducted after pretreatment with DPA for 2 h and KA injection. (**A-C**) Effects of DPA (0.5 mg/kg) on the seizure score, the number of seizure within 90 min and the seizure duration, respectively, in KA-treated mice. Seizure evaluation was carried out after KA injection. All the data were expressed as mean ± SEM (*n*=6). **p*<0.05, ****p*<0.001. *Abbreviation*: DPA, D-penicillamine; KA, kainic acid.

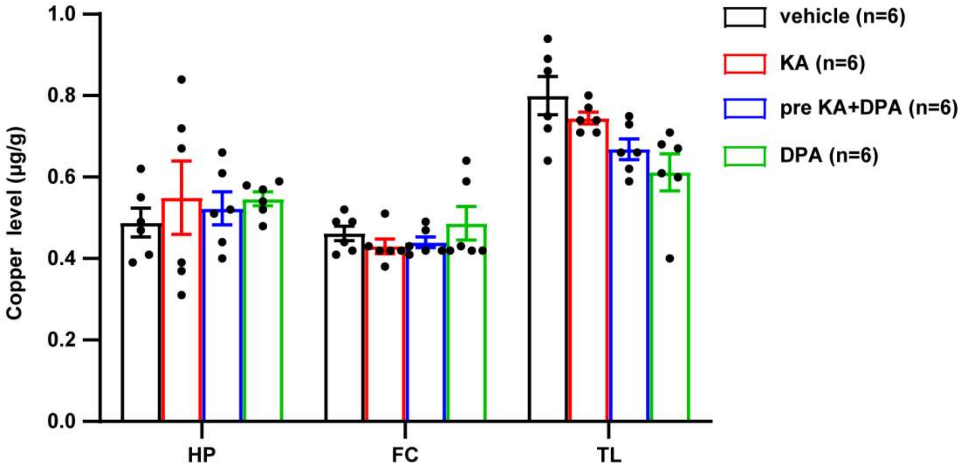

Figure S2. DPA does not alter the copper level in the brain tissues of KA-treated mice. Tissue samples from different brain regions including hippocampus, frontal cortex and thalamus were collected for detection of copper level following pretreatment with DPA (0.5 mg/kg) for 2 h and 3 d after KA (250 ng/μL) injection. All the data were expressed as mean ± SEM (*n*=6). *Abbreviation*: DPA, D-penicillamine; KA, kainic acid; HP, hippocampus; FC, frontal cortex; TL, thalamus.

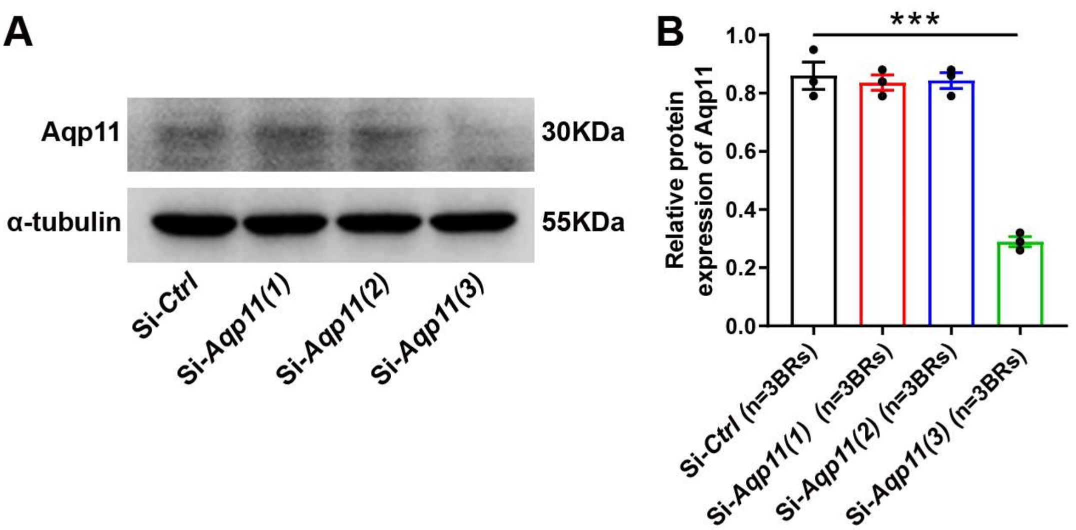

Figure S3. Si-*Aqp11 (3)* evidently decreases the protein expression of Aqp11 in HT22 cell. After transfection with three different si-RNAs of Aqp11 or matched control for 48 h, cell samples were collected for evaluating knock down efficiency by detecting Aqp11 protein expression. It was noted that transfection of si-*Aqp11 (3)* exhibited the most evident reduction of Aqp11 protein level. Thus, si-*Aqp11 (3)* was selected for the subsequent experiments. (**A-B**) show the representative blot images and statistical analysis. All the data were expressed as mean ± SEM (*n*=3 independent biological replicates). ****p*<0.001. *Abbreviation*: BRs, biological replicates.

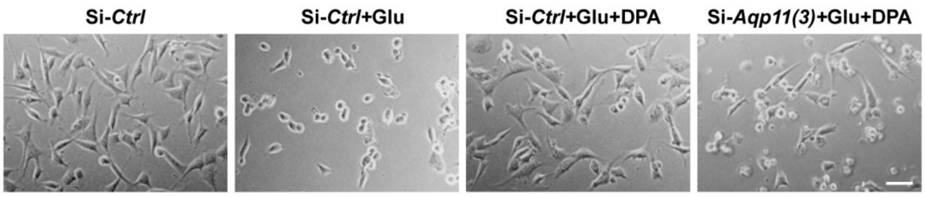

Figure S4. Knockdown of Aqp11 by siRNA transfection significantly blocks the protection of DPA against glutamate-induced cell death in HT22 cells. After transfection with three different si-RNAs of Aqp11 or matched control for 48 h, HT22 cells were cultivated in 24-well plate for 12 h. Then, DPA was pretreated for 2 h and subject to glutamate for another 8 h. Phase contrast images from each group were captured after glutamate challenge for 8 h. Scale bar indicates 50 μm. *Abbreviation*: DPA, D-penicillamine; Glu, glutamate.

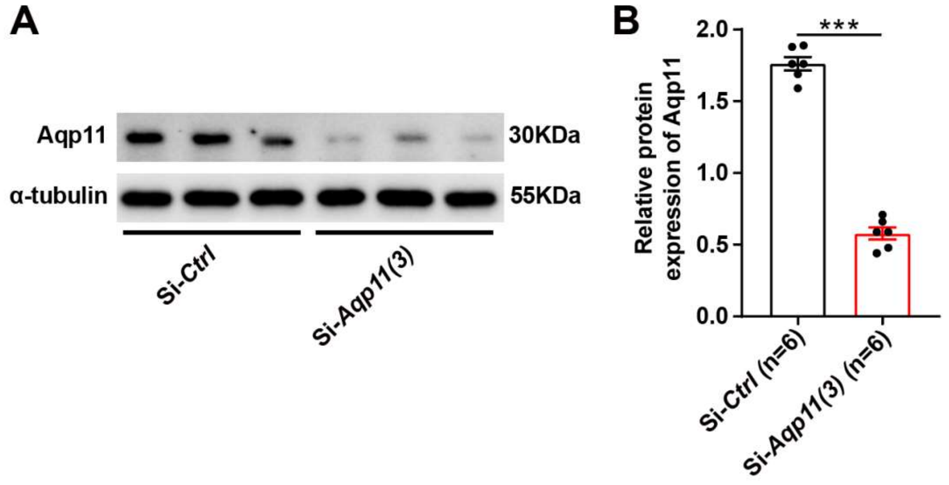

Figure S5. Si-*Aqp11 (3)* administration in vivo significantly results in the reduction of Aqp11 protein expression in mice hippocampus. Tissue samples were collected for western blot analysis after in vivo si-*Aqp11 (3)* administration for 48 h. (**A-B**) indicate the representative blot images and statistical analysis. All the data were expressed as mean ± SEM (*n*=6). ****p*<0.001.
